# Modelling diverse sources of *Clostridium difficile* in the community: importance of animals, infants and asymptomatic carriers

**DOI:** 10.1017/S0950268819000384

**Published:** 2019-03-18

**Authors:** A. McLure, A. C. A. Clements, M. Kirk, K. Glass

**Affiliations:** 1 Research School of Population Health, Australian National University, Canberra, Australian Capital Territory, Australia; 2 Faculty of Health Sciences, Curtin University, Perth, Western Australia, Australia

**Keywords:** *Clostridium difficile*, community-acquired infection, hospital-acquired infection, mathematical disease model, zoonotic infection

## Abstract

*Clostridium difficile* infections (CDIs) affect patients in hospitals and in the community, but the relative importance of transmission in each setting is unknown. We developed a mathematical model of *C. difficile* transmission in a hospital and surrounding community that included infants, adults and transmission from animal reservoirs. We assessed the role of these transmission routes in maintaining disease and evaluated the recommended classification system for hospital- and community-acquired CDIs. The reproduction number in the hospital was <1 (range: 0.16–0.46) for all scenarios. Outside the hospital, the reproduction number was >1 for nearly all scenarios without transmission from animal reservoirs (range: 1.0–1.34). However, the reproduction number for the human population was <1 if a minority (>3.5–26.0%) of human exposures originated from animal reservoirs. Symptomatic adults accounted for <10% transmission in the community. Under conservative assumptions, infants accounted for 17% of community transmission. An estimated 33–40% of community-acquired cases were reported but 28–39% of these reported cases were misclassified as hospital-acquired by recommended definitions. Transmission could be plausibly sustained by asymptomatically colonised adults and infants in the community or exposure to animal reservoirs, but not hospital transmission alone. Under-reporting of community-onset cases and systematic misclassification underplays the role of community transmission.

## Introduction

*Clostridiodes difficile*, more commonly known as *Clostridium difficile*, is an emerging pathogen that causes potentially life-threatening diarrhoea and is increasing in burden in many parts of the world [1–3]. In the USA, it caused an estimated 453 000 infections and contributed to 29 300 deaths in 2011 [3]. *C. difficile* infections (CDIs) are common in healthcare facilities where they account for 71% of hospital-associated gastrointestinal infections [4], but there is increasing recognition of community-acquired cases and healthcare-acquired cases with onset of symptoms in the community [3]. It is likely that many CDIs in the community go unreported, either because affected people do not seek treatment [5], do not submit a stool sample when they seek treatment [5] or their stool sample is not tested for *C. difficile* when submitted [6]. However, the extent of under-reporting has not known.

Colonised infants [7–10], contaminated food [11] and animals reservoirs [12] have been identified as possible sources of *C. difficile* outside hospitals, however their contribution to transmission has not been well quantified. Infants under 12 months have much higher prevalence of colonisation than adults [13], can be colonised for over 6 months by a single strain [7] and rarely develop symptoms but shed the same density of spores in their faeces as adults with CDI [8]. However, existing models of *C. difficile* do not capture infant colonisation or their potential role in transmission. Some strains of toxigenic *C. difficile* that cause disease in humans are also isolated from livestock, meat and fresh produce contaminated by animal faeces [11, 12]. However, the proportion of human cases that are acquired from food or animals and the ramifications for disease control are unknown.

The Infectious Disease Society of America (IDSA) and the Society for Healthcare Epidemiology of America (SHEA) recommend that CDI cases be classified as community-acquired or hospital-acquired according to time between onset of symptoms and most recent hospital admission or discharge [14]. Though the recommended system is not evidence-based [14], the system and minor variants are widely used to estimate the incidence of hospital- and community-acquired cases in the USA and many other countries [2, 3, 15, 16]. The recommended classification system has been shown to incorrectly classify many CDIs amongst hospitalised patients, underestimating the proportion of cases acquired prior to hospitalisation [17]. However, there has been no published assessment of the full classification system as applied to hospital-onset and community-onset cases.

Despite the importance of the community as both a source of new infections and the location of onset for some healthcare-associated infections, there is to date only one published model of *C. difficile* transmission that explicitly models patients outside hospitals [18]. The same model estimates an upper bound to the transmission from food and animals but does not explore the consequences of animal exposure as a source of *C. difficile* transmission. There have been no models that include the potentially important role of infants. We developed a model of *C. difficile* transmission in hospitals and communities to explore the contributions of hospitals, communities, adults, infants, animals and food to the transmission of toxigenic *C. difficile* in human populations. We also estimated the extent of under-reporting in the community and assessed the commonly used definitions of hospital- and community-acquired CDI.

## Methods

### Model structure

**Fig. 1.**
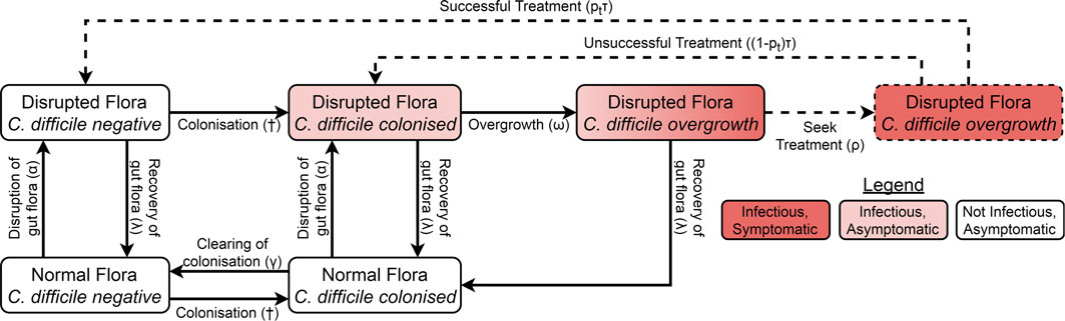
Model structure showing including colonisation, gut flora status, symptoms and treatment. Adults in the immune classes do not have symptoms and therefore not all individuals with overgrowth seek or receive treatment (dashed arrows and box). The details for infants, immunity, demographics and hospital–community structure are summarised in Figure S1. The definitions and values of the parameters associated with each transition can be found in Table S1. †The force of colonisation depends in the number and type of infectious individuals in the same setting (hospital or community).

We adapted a compartmental model of toxigenic *C. difficile* transmission in hospitals [19] to model transmission in a hospital and the surrounding community, adding treatment seeking, compartments for infants under 12 months, demographic processes, waning immunity and transmission from animal reservoirs. The model of the non-infant population had the same structure in both the hospital and community, with non-infants distributed amongst different compartments according to their immunity to *C. difficile* toxins, *C. difficile* colonisation state and the state of their gut flora. However, antibiotic prescription rates and treatment-seeking behaviour differed between the hospital and community while infants were only modelled in the community. The structure is summarised in Figure 1 and Figure S1.

Non-infants (whom we call adults from here on) had three immune statuses: able to mount an effective immune response to *C. difficile* toxins conferring resistance or immunity to symptoms but not colonisation; naive to *C. difficile* toxins but with a healthy immune system; and unable to mount an effective immune response to *C. difficile* toxins because of advanced age or a suppressed immune system. Immunity could be conferred to any non-suppressed adult by either extended asymptomatic carriage or recovery from CDI. Any immune person could have their immunity wane when they are not colonised and any non-suppressed individual (including infants) could age to become suppressed.

There were two possible commensal gut flora statuses for adults: disrupted and not disrupted. There were four possible *C. difficile* statuses: free of *C. difficile*, colonised, *C. difficile* overgrowth without treatment and *C. difficile* overgrowth with treatment. As we were concerned primarily with strains that can cause symptomatic disease, we only modelled toxigenic strains of *C. difficile*. An individual could have almost any combination of gut flora and *C. difficile* statuses, but we assumed that *C. difficile* overgrowth could only occur in individuals with disrupted gut flora. Non-immune adults with *C. difficile* overgrowth were considered symptomatic, while all other colonised individuals (infants, immune adults and adults without *C. difficile* overgrowth) were considered asymptomatic. Both symptomatic and asymptomatically colonised individuals shed spores and so were infectious [20]. Spore shedding has been observed to increase before toxin production [21], but decrease during *C. difficile* treatment [22]. Therefore, asymptomatically colonised individuals with disrupted gut flora and individuals with overgrowth were equally infectious, patients receiving treatment had reduced infectiousness determined by the effectiveness and coverage of contact precautions [19] and colonised patients with intact gut flora transmitted at a reduced rate.

Since CDI is only rarely observed in infants under 12 months and antibiotics do not predispose infants to carriage [10], the model for infants was much simpler than for adults, consisting of only three compartments. At birth, infants were not colonised [8, 9] and did not have immunity [23]. As with adults, colonisation conferred immunity, but for simplicity we assumed this occurred immediately so there was no colonised-but-not-immune class for infants. Infants could clear their colonisation [8, 9]. Infants aged by entering the corresponding adult class with intact gut flora that shared the same colonisation and immune states.

### Model parameterisation

Many of the parameters used in this model were based on our previous model of *C. difficile* transmission in hospitals [19] and/or drawn from the literature (Table S1). Eight parameters were fitted to data in this study. The likelihood function used to fit the model was composed from data for the prevalence of colonisation [9] and immunity [23] at given ages, longitudinal infant colonisation [8, 9], the proportion of hospital admissions with CDI as the primary diagnosis [24] and the incidence of reported hospital- and community-acquired cases [3]. The reported estimates of the prevalence of toxigenic colonisation in the general adult population have varied considerably between settings and studies. These studies have used different detection methods and often had small sample sizes [13]. Therefore, we considered multiple scenarios with colonisation prevalence from the range 2–10%, with a default of 5%. We determined the values of the eight parameters that (A) ensured that a predetermined proportion (in the range 2–10%) of the general adult population was colonised and (B) maximised the model likelihood. This was repeated for a range of values of the colonisation prevalence in the general adult population. See Supplementary materials for details of all parameters and how they were estimated.

### Transmission from infants

Despite their high carriage rates, there has been little research on the contribution of infants to *C. difficile* transmission. Furthermore, the relative infectiousness of infants and adults in the community cannot be determined using our model and available data. It has been shown that the mean density of *C. difficile* per gram of stool is similar for asymptomatically colonised infants and adults with CDI [8]. However, many other unquantified factors (e.g. hygiene practices and the number of social contacts) contribute to infectiousness, so we considered a wide range of assumptions in our sensitivity analysis. In a preliminary analysis, model fit was poor and/or the proportion of transmission from infants implausibly high in scenarios where infant infectiousness exceeded that of symptomatic adults. Therefore, we considered relative infant infectiousness in the range 0–1 for our sensitivity analysis with 0.5 as conservative default assumption.

### Accounting for under-reporting and misclassification of CDIs

To fit our model to incidence estimates for CDI [3], we simulated the processes of treatment seeking, reporting and the classification of cases as hospital or community-acquired. We assumed that, as with other diarrhoeal diseases, some patients recover from CDI without seeking treatment [25], by modelling treatment seeking in the community and recovery as competing hazards (see Supplementary materials for details). To account for the low testing rate for diarrhoea in general [5, 25] and community-onset CDI in particular [6], we estimated the proportion of cases seeking treatment in the community that were identified, allowing us to compare model outputs to published estimates of disease burden based on notification data [3].

**Fig. 2.**
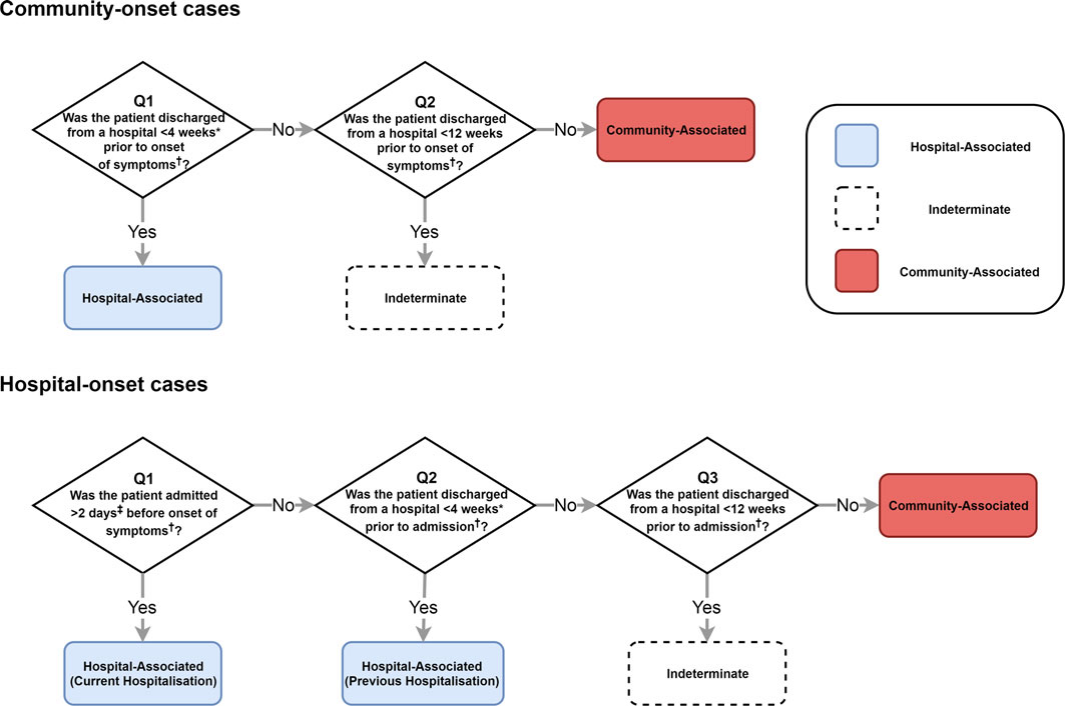
The classification of CDI cases based on IDSA and SHEA surveillance recommendations that we assessed with our model. Lessa *et al*. used a similar classification scheme to estimate incidence in the USA. *Lessa *et al*. used a 12-week cut-off and therefore do not classify any cases as ‘indeterminate’. ‡Lessa *et al*. used a 3-day cut-off. †We used symptom onset or hospital admission as reference points in our simulations. However, the classification system recommended by IDSA and SHEA uses onset of symptoms as the reference point for all cut-offs. Our classification is otherwise identical. Lessa *et al*. used date of positive faecal sample as reference point.

The IDSA and SHEA recommend surveillance definitions that classify where a CDI was acquired by location of onset of symptoms (healthcare facility or community) and by time since the most recent hospital discharge or admission [14] (Fig. 2). Lessa *et al*. [3] employed a variant of these definitions to estimate the incidence of initial (i.e. non-recurrent) hospital- and community-acquired CDIs in the USA (Fig. 2). We therefore emulated this classification system to fit our model to the incidence of hospital- and community-acquired CDIs reported by Lessa *et al*. (see Supplementary materials for further details).

To determine the true origin of an infection in our model, we subdivided each *C. difficile*-positive compartment into hospital-acquired and community-acquired compartments, allowing us to track where infection was acquired even if patients moved between settings once or more between acquisition and onset of symptoms. For simplicity, we assumed that current hospital-acquired colonisation prevented community-acquired colonisation and *vice versa*. This assumption had no effect on overall transmission dynamics. Moreover, coinfection with multiple strains (which may have been acquired from multiple sources) accounts for only approximately 10% of infections [26], so our simplifying assumption was unlikely to substantially affect the classification of infections. For each set of surveillance definitions, we calculated the sensitivity and precision to identify hospital- and community-acquired cases amongst both hospital-onset and reported community-onset cases, using the true origin of infection in our model as a gold standard. We identified cut-offs that improved on the existing definitions amongst reported cases, considering classification systems with a single cut-off for time since hospital admission and a single cut-off for time since most recent hospital discharge (i.e. classifying no cases as indeterminate). The *balanced* pair of cut-offs had equal sensitivity to identify hospital- and community-acquired cases amongst both hospital-onset cases and community-onset cases. The *optimal* pair of cut-offs had equal precision and sensitivity when identifying hospital-acquired cases, amongst both hospital-onset and community-onset cases.

### Reproduction number

Since the extent of human exposure to animal reservoirs of *C. difficile* is unknown, we calculated reproduction numbers assuming that all exposure was due to person-to-person transmission – an upper bound for the true reproduction number. We calculated the reproduction number for the whole population. We also calculated reproduction numbers for the community and the hospital separately. The latter calculations were identical to standard next-generation matrix calculations [27], except we only considered the colonised individuals in the setting of interest to be colonised for the purposes of the calculation. The reproduction numbers for hospital and community were the endemic threshold parameters in each setting assuming no external sources of *C. difficile* (movement of patients or animal reservoir).

### Food- and animal-driven transmission

The extent of zoonotic or foodborne *C. difficile* exposure is unknown; however, we considered the implications of differing amounts transmission from animal reservoirs. For a given force of colonisation, higher human exposure from food or animals implies less person-to-person transmission and therefore a smaller reproduction number. If a sufficient proportion of exposure originates from food or animals, the reproduction number in the human population is less than one and human disease is sustained by constant exposure to non-human sources of *C. difficile*. In this case, we say that *C. difficile* is *animal-driven*.

For each set of modelling assumptions, we calculated the extent of foodborne exposure that implied *C. difficile* was animal-driven. We expressed this *animal-driven threshold* in terms of exposures leading to colonisation per person per year and as a proportion of all transmission (i.e. foodborne transmission *and* person-to-person transmission).

## Results

### Model fit

The model fitted the data well, reproducing the observed age profile of toxigenic *C. difficile* colonisation, immunity, reported incidence of infection and proportion of admissions for CDI (Figure S2). For most scenarios, infant infectiousness did not affect model fit. However, the model fit was poor for combinations of low colonisation prevalence amongst adults and high infant infectiousness, so these scenarios were not considered further. The model was verified by outcomes not used to fit the model such as recurrence proportion for hospital and community cases, the proportion of hospital-based transmission attributable to symptomatic carriers, the duration of colonisation in infants and the greater proportion of elderly and immune suppressed in hospital-acquired *vs.* community-acquired cases (see Supplementary materials for details). In our model, colonisation prevalence was 17% higher (range: 4–55%) at hospital discharge than in the general adult population, agreeing with the common observation that colonisation is more common amongst those who have been recently discharged from hospital. However, 78% (range: 60–87%) of colonised discharges had acquired the pathogen in the community prior to admission and remained colonised for the duration of their hospital stay. We estimated a mean immune period of 9.4 years (range: 4.0–30.4 years) with the longest immune period when we assumed adult colonisation prevalence was low (2%).

### Reproduction number and food-driven threshold

**Fig. 3.**
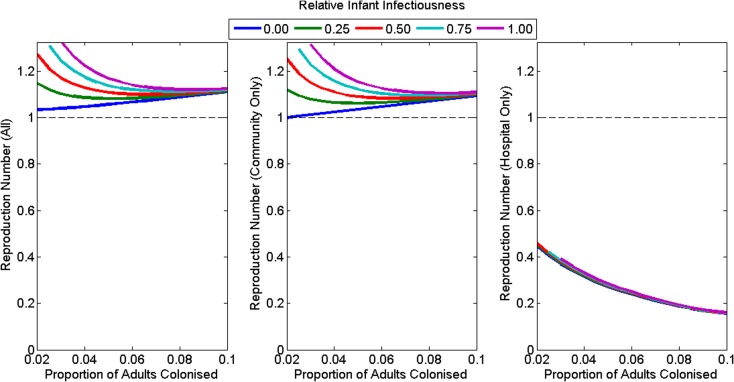
The reproduction number at the disease-free equilibrium for various plausible assumptions for the colonisation prevalence in adults and relative infectiousness of infants for (a) the whole population, (b) the community only and (c) the hospital only. The model had poorer model fit for the combination of high infant infectiousness and low adult colonisation prevalence, so these combinations are omitted from the figures.

Under the assumption of no foodborne transmission, the reproduction number for the whole population was greater than one for all plausible assumptions (default: 1.11, range: 1.03–1.35) (Fig. 3a). The reproduction number for the hospital was less than one for all plausible assumptions (default: 0.28, range: 0.16–0.46), decreasing with increasing colonisation prevalence of adults in the community and unaffected by assumptions concerning the infectiousness of infants (Fig. 3c). The reproduction number for the community was close to but lower than the reproduction number for the whole population (default: 1.09, range: 0.999–1.34) (Fig. 3b) and increased with increasing infant infectiousness. The reproduction number was less than one in the community only if infants were not infectious and adult colonisation prevalence was 2%.

**Fig. 4.**
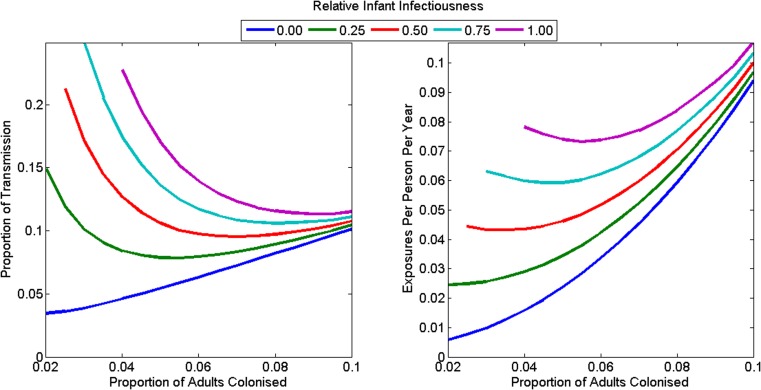
The animal-driven threshold under various plausible assumptions for the *C. difficile* colonisation prevalence in adults, and the relative infectiousness of infants as (a) a proportion of all transmission in the community and (b) as rate of exposure to adults in the community. The reproduction number is less than one in the community if transmission from animals exceeds the animal-driven threshold. The model had poorer model fit at the animal-driven threshold for the combination of high infant infectiousness and low adult colonisation prevalence, so these combinations are omitted from the figures.

The animal-driven threshold (the minimum force of colonisation attributable to food and animals that implies the reproduction number in the human population is less than one), was 0.046 exposures per person per year (range: 0.006–0.107) or 10.6% of all transmission in the community (range: 3.5–26.0%) (Fig. 4). This is equivalent to one foodborne or animal exposure leading to colonisation every 21.7 years per person (range 9.4–175.5 years). The animal-driven threshold was lowest (once every 175.5 years per person) if infants were not infectious and adult colonisation prevalence was low (2%). The animal-driven threshold was highest (once every 9.4 years per person) if infants were as infectious as adults and adult colonisation prevalence was high (10%). The model had poor model fit at the animal-driven threshold when infant infectiousness was high and adult colonisation prevalence was low.

### Transmission from infants and asymptomatic adults with intact gut flora

In our main analysis, 13–30% of transmission in hospitals was from patients receiving treatment for CDI, but <10% of all transmission in the community was attributable to symptomatic patients or patients with disrupted gut flora. The remaining transmission was attributable to infants or asymptomatically colonised adults with intact gut flora. The proportion of transmission in the community attributable to infants was 17.4% for our conservative default scenario but was highly sensitive to the relative infectiousness of infants and colonisation prevalence in adults (Figure S3). With infants as infectious as symptomatic adults and adult colonisation prevalence in the community at ⩽5%, ⩾40% of transmission in the community was attributable to infants. The proportion of transmission attributable to asymptomatically colonised individuals with intact gut flora was also highly sensitive to these assumptions (Figure S4). Under default assumptions, this group accounted for 79% of transmission in the community and 25% of transmission in the hospital, but ⩾90% of transmission in the community if colonisation prevalence was 10% amongst adults in the community. Patients with CDI and colonised individuals with disrupted gut flora were 6.6 times more infectious (range: 2.8–131.8) than colonised individuals with intact gut flora, but were much less numerous, especially in the community where the antibiotic prescription rate was low. Infants cleared their colonisation 9.2–11.5 times more slowly than adults with intact gut flora. Under most scenarios, infants were also more exposed or susceptible to colonisation (default: factor of 1.4; range: 0.6–4.4) and more infectious (default: factor of 3.3; range: 0–9.8) than asymptomatic adults with intact gut flora (compare Table 1 and Table S1).

**Table 1.** Definitions, values and references for eight parameters fitted with the model

Parameter	Description	Value (Sensitivity analysis range)
*σ*	Rate at which immunity wanes	2.9 × 10^−4^ (0.9–6.9 × 10^−4^)
*θ*	Multiplicative factor for colonisation susceptibility of infants	1.4 (0.6–4.4)
*γ*_infant_	Rate at which *C. difficile* is cleared in infants	2.0 × 10^−3^ (2.0–2.5 × 10^−3^)
*p*_disrupt_	Proportion of antibiotics that disrupt gut flora	0.22 (0.12–0.48)
*p*_report_	Proportion of all community-treated CDIs that are reported	0.63 (0.57–0.76)
*ν*_CDI_	Hospital admission rate for CDI	1.4 × 10^−2^ (1.3–1.7 × 10^−2^)
*β*_Disrup*t*_	Transmission rate coefficient for colonised adults with disrupted gut flora (due to recent antibiotic exposure)	12.8 × 10^−2^ (7.1–17.4 × 10^−2^)^a^
*β*_Intact_	Transmission rate coefficient for colonised adults with intact gut flora (no recent antibiotic exposure)	1.9 × 10^−2^ (0.1–2.6 × 10^−2^)^a^
*β*_infant_	Transmission rate coefficient from infants	6.4 × 10^−2^ (0–17.4 × 10^−2^)^a^

A full list of parameters can be found in Table S1. All rates are in units of day^−1^.

a Only these parameters were affected by assumptions around infant infectiousness, being estimated under the assumption that *β*_Infant_ = *k* *×* *β*_Disrupt_ for *k* in the range 0–1.

### Under-reporting and misclassification of CDIs

Though we estimated that patients with CDI were admitted to hospital at 59 (range: 53–73) times the rate of the general adult population (Table 1), only 48% of adults with community-onset CDIs sought treatment in the community or hospital (Table 2) and only 63% (range 56–76%) of CDIs treated in the community were reported (Table 1). Therefore, while we assume that 100% of symptomatic hospital-onset infections were reported, we estimate that only 30% (range 27–37%) of all community-onset CDIs were reported. Considering both hospital- and community-onset CDIs, only 67% (range 66–70%) of all hospital-acquired CDIs and 35% (range 33–40%) of all community-acquired cases were reported (Table 2).

**Table 2.** Simulated incidence of hospital-acquired (HA) and community-acquired (CA) CDIs, under-reporting of cases and classification errors for two different simulated classification schemes

		Actual Proportion of all reported cases %	IDSA/SHEA recommendations			Lessa *et al*., classification		
	Proportion reported %		Proportion of all reported cases % ^b^	Precision %	Sensitivity %	Proportion of all reported cases % ^c^	Precision %	Sensitivity %
Any HA	67 (66–70)	15 (9–23)	38 (38–39)	37 (24–57)	97 (96–97)	41	34 (22–53)	94 (93–95)
Any CA	35 (33–40)	85 (77–91)	57 (56–57)	99 (99–100)	65 (61–72)	59	99 (97–99)	68 (64–74)
HO-HA	100^a^	11 (7–17)	25 (25–25)	44 (29–65)	97 (96–97)	23	46 (31–67)	93 (91–94)
HO-CA	100^a^	17 (11–21)	3 (3–3)	89 (77–94)	15 (14–19)	5	85 (70–92)	27 (25–33)
CO-HA	31 (28–37)	3 (2–6)	13 (13–14)	24 (15–42)	96 (95–97)	18	18 (11–33)	99 (99–99)
CO-CA	30 (27–36)	69 (66–70)	54 (53–54)	>99.8	78 (76–81)	53	>99.8	78 (76–81)

HA, hospital-acquired, CA, community-acquired, HO, hospital-onset, CO, community-onset.

The range in parenthesis is the range across all sensitivity analysis scenarios. Classification sensitivity is amongst reported cases only; thus, multiplying by the reported proportion will return the sensitivity amongst all cases.

a We assumed that all hospital-onset infections are reported.

b Percentages do not sum to 100% as some cases are classified as ‘indeterminate’ under this system.

c The model was fit to estimates of CDI incidence that used this scheme to classify the location of acquisition. Consequently, these values change very little across the sensitivity analysis scenarios.

**Fig. 5.**
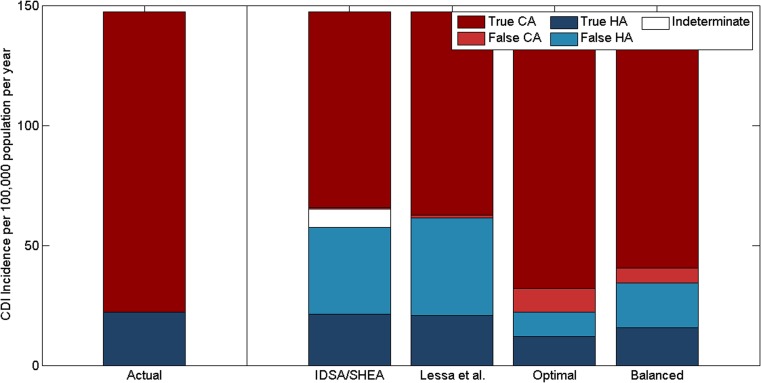
Classification of the origin of reported CDIs by time since hospital discharge or admission, comparing the actual incidence of reported hospital-acquired (HA) and community-acquired (CA) CDIs *vs*. the classification recommended by IDSA and SHEA and three variants. Lessa *et al*. use a 3-day cut-off for recent hospital admission and a 12-week cut-off for recent hospital discharge. The optimal and balanced classifications we have identified use 7.4- and 6.6-day cut-offs, respectively, for recent hospital admission and 2.1- and 12.5-day cut-offs, respectively, for recent hospital discharge.

Standard CDI classification schemes misclassified many of the reported community-acquired cases as hospital-acquired in our model: 63% (range: 43–76%) of cases classified as hospital-acquired with the IDSA/SHEA scheme were actually community-acquired (Table 2). The classification systems were much more precise but less sensitive for community-acquired cases (Table 2). Though total incidence was underestimated due to under-reporting, both classification schemes overestimated the proportion of reported cases that were hospital-acquired (Fig. 5). We estimate that only 40% (range: 26.5–60.6%) of hospital-onset and 4.5% (range: 2.7–8.4%) of reported community-onset infections are hospital-acquired. In contrast, the classification scheme recommended by IDSA and SHEA classified 89.6% (range: 88.9–90.3%) of hospital-onset and 19.6% (range: 19.4–20.3%) of reported community-onset infections as hospital-acquired. A 7.4-day cut-off (range: 5.0–9.5) for recent hospital admission (in hospital-onset cases) and a 2.1-day cut-off (range: 1.3–3.9) for prior hospital discharge were the optimal pair of cut-offs. A 6.6-day cut-off (range: 5.8–7.0) for recent hospital admission and a 12.5-day cut-off (range: 11.8–14.5) for prior hospital discharge were the balanced pair of cut-offs. The optimal cut-off correctly estimated the proportion of cases that were hospital- or community-acquired, but had poor precision (≈50%) to identify hospital-acquired cases (Fig. 5).

## Discussion

Under all reasonable scenarios and modelling assumptions, transmission between hospitalised adults amplified disease burden (higher force of colonisation and higher colonisation proportion in discharged patients than the general population) but was not the key driver of toxigenic *C. difficile* transmission in the population (hospital reproduction number less than one), in agreement with previous modelling studies [19, 28]. When we simultaneously assumed low prevalence of *C. difficile* colonisation in adults, no infant infectiousness and no transmission from non-human sources, the reproduction number in the community was also less than one. In this unlikely scenario, the movement of colonised individuals between hospital and community was essential for persistence of *C. difficile* in both settings. However, in all other scenarios without transmission from non-human sources, the reproduction number was greater than one in the community, and therefore transmission in the community would persist even in the absence of transmission in hospitals. This is the first time reproduction numbers have been estimated for *C. difficile* in a model including both the hospital and the community.

Symptomatic carriers of *C. difficile* accounted for <10% of transmission in the community in our model. Despite accounting for <2% of the total population, infants under 12 months accounted for 17% of transmission in the community for our conservative default assumptions and ⩾40% of transmission if infants were at least as infectious as symptomatic adults and colonisation prevalence was ⩽5% in the community. However, the exact proportion was highly sensitive to the relative infectiousness of infants (which has not been well quantified) and the colonisation prevalence in adults in the community (which varies considerably between studies and settings [13]). Nevertheless, our results indicate that asymptomatically colonised infants are likely to be a substantial source of transmission in the community. This is in agreement with a number of small studies that found CDI was associated with exposure to infants [29, 30] and a large study that, despite sampling only 1% of infants in Oxfordshire, was able to determine that 2% of all known CDIs in Oxfordshire could be reasonably attributed to recent direct or indirect transmission from these infants [31].

We investigated how transmission from non-human sources affected estimates of the reproduction number for person-to-person transmission. We demonstrated that the reproduction number in the human population was less than one if over 3.5–26.0% of transmission in the community was from non-human sources such as food or water contaminated by livestock animals. If current transmission from animals is above this threshold, *C. difficile* could not persist in the human population without these non-human exposures. This animal-driven threshold in terms of *C. difficile* exposures per person per year was remarkably low: equivalent to one exposure leading to colonisation per adult every 21.7 years under our default assumptions. For comparison, it has been estimated that Australians have an episode of foodborne gastroenteritis (i.e. not counting asymptomatic exposure) on average once every 5 years [32]. Given the overlap of strains between animals and humans, the presence of *C. difficile* spores on raw meats and fresh vegetables [11], and the high survival rate of *C. difficile* spores following cooking at recommended ‘safe’ temperatures [33], it is plausible that exposure exceeds this low threshold. Though our model has not accounted for multiple strains of *C. difficile*, one could apply the animal-driven threshold to individual strains or types of *C. difficile*. For instance, it is not plausible that ribotype 001, which accounts for a substantial proportion of human cases but is not common in food animals [34], exceeds the animal-driven threshold. On the other hand, it is plausible that ribotypes 078, 027 and other ribotypes that both cause human infection and are commonly isolated from animals [34], exceed the threshold. This is especially true for ribotype 078, as isolates from humans and food animals appear to be closely related [12].

We estimate that approximately 70% of community-onset cases are not reported, either because the patient does not seek treatment, or the pathogen remains unidentified. This is in agreement with the low treatment-seeking rates reported generally for diarrhoea [5, 25] and low testing rates for *C. difficile* in primary care [6]. The simulated proportion of community-acquired cases was higher amongst community-onset cases than hospital-onset cases. Consequently, we estimate that while two-thirds of hospital-acquired infections are reported, only one-third of community-acquired infections are reported. Though we only simulated the under-reporting of community-onset cases, our findings complement an empirical study that found that missed cases of CDI in hospital settings are disproportionately likely to be community-acquired [35]. Existing classification schemes attempt to account for cases that may acquire the pathogen in one setting and, after an incubation period, develop symptoms in another; however, these schemes are highly asymmetric with regards to setting [3, 14]. While only hospital-onset cases with symptom onset within 2 or 3 days of hospital admission are considered community-acquired, all community-onset cases with symptom onset within 4 or 12 weeks of hospital discharge are classified as hospital-acquired. Empirical estimates of the median incubation period vary considerably from 18 to 33 days [36] but lie between the two extremes of these cut-offs. Therefore, it is likely that typical cut-offs for classifying hospital-onset cases as community-acquired are too short and typical cut-offs for classifying community-onset infections as hospital-acquired are too long. We confirmed this with our model by demonstrating that, to balance the sensitivity and specificity of classification, these cut-offs should be approximately 6 and 12 days, respectively. We also demonstrated that any scheme based on time since most recent hospital discharge cannot adequately distinguish hospital- and community-acquired cases. Even our balanced scheme had very poor precision for hospital-acquired cases: half or more of all cases classified as hospital-acquired were actually community-acquired. This can be understood by noting that the mean length of hospital stay (between 4 and 10 days in most high-income countries [37]) is short compared with the duration of colonisation, which may be several weeks [38]. Consequently, more than 60% of patients who were colonised at hospital discharge in our model were not exposed in the preceding hospitalisation, but rather in the community prior to admission. Therefore, even patients who developed CDI very soon after hospital discharge were more likely to be community-acquired than hospital-acquired. Adjusting the cut-off times cannot correct this flaw in the existing classification schemes. Alternative schemes, such as classification based on the total number of days spent in hospital in the weeks leading up to the onset of symptoms, should be considered. Our model demonstrates that the classification scheme recommended by IDSA and SHEA has very high sensitivity for hospital-acquired cases, and therefore may be useful if all hospital-acquired cases need to be identified or excluded. However, the asymmetry and unrealistic timescales in existing classification schemes inadvertently reinforce the *a priori* assumptions upon which they are based: that the colonisation pressure in hospitals far exceeds the colonisation pressure in the community.

Our study has several limitations. The data used to fit the model were incomplete and were gathered from many different sources, countries and settings. In particular, the published estimates of colonisation prevalence vary significantly between studies and it is unclear to what extent this reflects genuine differences between study populations or variations associated with different detection methods or small sample sizes [13]. We addressed this by considering a range of scenarios that reflected the possible range of colonisation prevalence. The relative infectiousness of infants and adults is also unknown, so we allowed the relative infectiousness of infants and adults to vary in our sensitivity analysis but were therefore unable to provide a precise estimate of the amount of transmission attributable to infants. However, our broad sensitivity analysis and wide variety of input data improve the global applicability of the model. National estimates in the USA suggest that nearly all people with CDI had received some form of healthcare soon before onset of symptoms if outpatient and primary care were included [3]. This does not imply that most CDIs are healthcare-acquired, since antibiotic exposure is a causative factor for CDI and antibiotics are prescription-only medicines in many countries, including the USA. However, we were unable to model pathogen acquisition from other sources of healthcare, because the hospital in our model consisted only of admitted patients, with the community including patients receiving all other forms of healthcare (including residents of long-term care facilities). Long-term care facilities contain sub-populations of individuals at high risk for CDI [3]. As we have not modelled long-term care facilities separately, we are likely to have underestimated the heterogeneity and thus the reproduction number in the community. The model population is well-mixed and does not capture heterogeneity in hospital admission rates or heterogeneity in the contact rates of infants, adults and the elderly, which may also affect reproduction number estimates. Finally, we did not differentiate between the many strains of *C. difficile* [39], so it is possible the hospital reproduction numbers, community reproduction numbers and animal-driven thresholds differ by strain.

Under-reporting of community-onset CDIs and the misclassification of many community-acquired infections obscure and underestimate the extent of transmission in the community. It seems likely that unreported community-onset cases will be less severe and that the classification (or misclassification) of individual cases as hospital-acquired or community-acquired will not affect the treatment or outcomes of patients. Therefore, at the level of individual cases, even large-scale under-reporting and misclassification may not be very harmful if those with severe disease receive appropriate care. However, to prevent infections, we must understand when, where and how transmission occurs at the level of the population. We have demonstrated that most infections (hospital-onset and community-onset alike) are acquired outside of hospitals, but only a small fraction are reported. Therefore, interventions that prevent acquisition outside hospitals, or prevent patients admitted with asymptomatic colonisation from developing symptoms should be considered and assessed. Merely reducing transmission between hospitalised patients will not be sufficient to prevent the spread of this important pathogen. Further investigation into the relative infectiousness of infants is required before the proportion of transmission from infants can be estimated. However, we have demonstrated that a high degree of transmission from infants is consistent with available data on spore shedding [8] and colonisation prevalence [8, 9]. Similarly, though the frequency of food and animal-to-human transmission is unknown for *C. difficile*, we have demonstrated that even very modest and plausible frequencies of exposure may imply that *C. difficile* is sustained in human populations by transmission from animals or contaminated food. If this is the case, *C. difficile* can be eradicated from the human population if and only if animal-to-human transmission is reduced.

## Supplementary material

For supplementary material accompanying this paper visit https://doi.org/10.1017/S0950268819000384. click here to view supplementary material
