# Supplementary material for: Modelling diverse sources of Clostridium difficile in the community: importance of animals, infants and asymptomatic carriers
Source: Epidemiol Infect. 2019 Mar 18;147:e152. doi: 10.1017/S0950268819000384 (PMC6518831; doi:10.1017/S0950268819000384)
Supplement: Supplementary file 1 [file S0950268819000384sup001.zip › Supplementary_Materials_1_-_Revised.docx]

# Epidemiology and Infection

# Modelling diverse sources of *Clostridium difficile* in the community: importance of animals, infants and asymptomatic carriers – Supplementary Material

Angus McLure, Archie C. A. Clements, Martyn Kirk and Kathryn Glass

## Further details of model structure and parameters

### Demographics

The demographic structure of the model is summarised in **Supp. Figure 1**. All individuals are born into the infant class without immunity or colonisation. We assume the population is closed without immigration or emigration. The birth rate is chosen to balanced deaths such that the equilibrium population is N_Comm_ (100,000). Infants age to become (non-suppressed) adults at rate ζ (ζ^-1^ = 1 year). Non-suppressed adults and infants age slowly to become suppressed adults at rate ψ such that the median time to age is 65 years ($\psi=\frac{\ln2}{65\times365}\mathrm{day}^{-1}$).

There are two death rates: one rate for infants and non-supressed adults (φ) and a much higher rate for supressed/elderly adults (φ_U_). The two death rates are chosen so that proportion in the suppressed class is 13.7% (the proportion of US population over 65 [1]) and proportion of deaths in the suppressed class is 72.4% (proportion of deaths that are in people over 65 in the US [1]): $\varphi_{U}=\psi\frac{1-0.137}{0.137}$ and $\varphi= \psi\frac{1-0.724}{0.724}$.

### Immunity

The immune structure of the model is summarised in **Supp. Figure 1**. Non-supressed adults, being treated for CDI are conferred immunity upon end of treatment. Colonised, non-supressed, adults not receiving treatment develop immunity at rate δ (1/ δ = 10 days) as determined in our previous model from sero-conversion rate in response to *C. difficile* toxoid vaccine trials [2,3]. To simplify the model infants develop immunity immediately on colonisation. All immune individuals have their immunity wane at rate σ, which is estimated with our model.

### Admissions and discharges

The hospital and community structure of the model is summarised in **Supp. Figure 1**. The hospital discharge rate for suppressed/elderly and non-supressed individuals is the inverse of the mean length of stay in US Hospitals for those over 65 (κ_U_ = 1/5.2 days) and those under 65 ( κ = 1/4.15 days) respectively (2012 data) [4]. However, those receiving treatment for CDI in hospital are not discharged. Similarly admission rates are determined from US hospital admission rates which are once every 11.4 years for those under 65 (ν) and once every 3.4 years for those over 65 (ν_U_) (2012 data) [4]. We assume that those who have symptoms of CDI in the community are admitted at a much faster rate (ν_CDI_) estimated with our model.

### Colonisation and Gut Flora

The model structure for gut flora disruption and *C. difficile* colonisation is summarised in **Figure 1** in the main text. Gut flora is disturbed at different rates in the hospitals and communities to reproduce the reported proportion admissions in hospitals [5] or proportion of adults in the community each year [6] that receive antibiotics. In the community those in the suppressed/elderly received antibiotics at a higher rate such that α_Comm_ < α_U_,_Comm_ < α_Hosp_ = α_U_,_Hosp_. The time take for the recovery of gut flora [7], and the duration of heightened risk of *C. difficile* following antibiotic exposure [8] depends on the antibiotic but we chose a single recovery rate λ = 0.03 day^-1^ which sits in the middle of the range. As it has been observed that 20% of hospitalised CDIs recover without specific treatment [9], the gut flora recovery rate for those with overgrowth is $\lambda_{o} =\frac{\rho_{Hosp}}{\frac{1}{0.2} -1}$.

The colonisation clearance rate is the same (γ) in all colonised adults and was determined in our previous model of *C. difficile* transmission in a hospital [10] based on the clearance rate in the control group in a trial for treating asymptomatic colonisation with vancomycin and metronidazole [11]. The clearance rate in infants (γ_infant_) is estimated in our model to the colonisation profile for infants. The rate at which *C. difficile* overgrows in those with damaged gut flora (*ω*) is the same in all adults and was determined for our previous model from observations of *C. difficile* overgrowth in a chemostat model of gut flora in human gastrointestinal tract [12].

### Transmission

Transmission is well mixed within each of the hospital and community but there is no transmission between these two locations (only movement of individuals), so there is a separate force of colonisation for the community and hospital. Person-to-person transmission comes from colonised adults with disrupted gut flora (β_Disrupt_), colonised adults with intact gut flora (β_Intact_) and from infants (β_infants_; community only). The transmission parameters from adults are estimated with our model. The base assumption is that infants are half as infectious as disrupted adults (β_infants_ = 0.5 β_Disrupt_), but we consider β_infants_ in the range 0 - β_Disrupt_ in our sensitivity analysis. Contact precautions (for patients receiving treatment) reduces transmission from these individual by factor q, determined for our previous model from contact precaution adherence rates [13]. (We assume no contact precautions in community, i.e. q_Comm_ = 1). Foodborne transmission adds to the force of colonisation in the community. Infants have different susceptibility to colonisation given by the factor θ. Therefore the force of colonisation for infants is θ times the force of colonisation in the community.

### CDI treatment and outcomes

The rate at which individuals with CDI seek treatment in the community was inferred from studies on treatment seeking behaviour for those with diarrhoea. Van Cauteren et al report that 33% of those who have diarrhoea seek treatment, with mean time to treatment seeking being 1.5 days [14]. Assuming a competing hazards model of treatment seeking and recovery, this means that the treatment seeking rate (ρ_Comm_) is 0.33/1.5 = 0.22 day^-1^. Treatment seeking rate in the hospital is much faster (ρ_Hosp_ = 1 day^-1^). Treatment rate (τ) is same in hospital and community (mean time is 10 days) [15]. Treatment success proportion (p_t_) is the same in hospital and community and was estimated in our previous model [10]. We assume that patients do not have a greater hazard of death with CDI as death due to CDI is sufficiently infrequent so as not to significantly affect population level outcomes.

## Details of parameter estimation

We used maximum likelihood estimation to determine the value of eight parameters. The likelihood function was composed of the product of likelihood functions for the colonisation prevalence in infants, the proportion seropositive for *C. difficile* toxin antibodies by age, the incidence of CDIs in hospitals and communities and number of patients admitted to hospital with CDI as a proportion of all admissions.

### Colonisation Prevalence in Infants and Proportion Seropositive by Age

Kubota et al (2016) collected stool samples from 111 Belgian neonates at approximately one, three, eight, 31, 91, 143 and 182 days after birth. Since the exact number of days since birth was not reported for each sample and infant, for the purposes of likelihood calculations we assumed that each set of samples was taken exactly at exactly one, three, eight, 31, 91 143 and 182 days after birth. Kubota et al. tested each sample for carriage of *C. difficile* and presence of genes for toxins A and B. They reported the sequence of sample results (including missing samples) for the 55 infants who had at least one *C. difficile* positive sample. For the 56 samples that had no positive tests (for which the sequence of negative and absent samples were not reported) we assumed that there were no missing samples and that all samples were negative. Since we are interested only in toxigenic *C. difficile,* we considered all *C. difficile* positive without either toxin genes, as negative samples.

Rousseau et al (2012) collected monthly stool samples from ten French infants in their first year of life starting at approximately one month. The exact number of days since birth were not given for each sample so we assumed that samples were taken (or were missed) at exactly 30-day intervals. They performed strain typing on each positive stool sample, typing either one or five colonies. They reported the sequence of sample results including typing and missing results. Since we are interested only in toxigenic *C. difficile*, we only considered a sample to be positive if at least one of the typed isolates was of a toxigenic strain.

Rousseau et al also collected a single stool sample from 85 French children aged 1.5-36.2 months from two day-care centres. They reported the number of samples positive for *C. difficile* and including the number positive for toxigenic strains of *C. difficile.* Again, we considered only the samples with a toxigenic strain to be positive and samples with non-toxigenic strains or no strain of *C. difficile* to be negative.

Holst et al. (1981) collected stool samples from 130 infants aged 1-12 months and 88 children aged 1-15 years (total 218 samples) and tested for *C. difficile* carriage. All *C. difficile* positive samples were for toxigenic strains. The proportion colonised was reported by monthly age brackets for infants under 12 months and larger brackets for older children. For likelihood calculations we assumed that the age of all subjects was exactly in the middle of their age bracket (measured in 30-day months).

Adlerberth et al. (2014) also reported longitudinal *C. difficile* colonisation data in infants, but did not provide denominator data, so their data were not used for fitting the model. However, they did demonstrate that infants can be long-term carriers toxigenic strains of *C. difficile,* finding that a third of colonised infants were still colonised by the same strain six months later.

Viscidi et al. (1983) report the age-related prevalence of antibodies to toxin A and toxin B of *C. difficile* in 98 paediatric in-patients and 242 outpatients from a US hospital. The trends for toxins A and B were similar. Since toxin A and B antibodies were measured from the same set of patients, the two trends cannot be considered independent, so we considered only the data for toxin-B prevalence.

The probability that an individual would be in each state (i.e. colonised/non-colonised or seropositive/seronegative) at a given age was calculated for a given set of parameters using the Markov chain approximation for an individual, with the force of colonisation at the equilibrium value. For the cross-sectional datasets, the model likelihood was derived from a product of binomially distributed random variables with n_i_ trials (the number of samples for age group *i*), x_i_ successes (i.e. the number of *C. difficile* positive or seropositive samples) and success probability p_i_ (the model predicted probability that an individual of that age group would be *C. difficile* positive or seropositive). For the datasets reporting the longitudinal sequence of sample results, the model likelihood a given sequence of results was the model probability that Markov chain of the individual would pass through those states at the sample ages.

The older, cross-sectional study by Holst et al. [16] found much higher prevalence of toxigenic colonisation amongst infants than recent studies [17,18], lead to poor model fit when combined with the recent studies or when used as the only infant colonisation data set and was therefore excluded.

### Incidence of healthcare-associated and community-associated CDI

Lessa et al. (2015) estimated the incidence of CDIs in the United States based on surveillance data from several US counties. They used a three-day cut-off for hospital onset CDIs and a single twelve-week cut-off for community onset CDIs to differentiate healthcare and community acquired CDIs. In their article they further subdivided healthcare associated cases into community-onset healthcare acquired, nursing-home onset and hospital-onset hospital-acquired cases. We simulated the application of the three-day and twelve-week cut-off to determine the model incidence of hospital-onset hospital-acquired CDIs, community-onset hospital acquired CDIs and community acquired CDIs. However, because our model did not differentiate between nursing home and the general community, we included nursing home onset cases in with community acquired cases for the construction of the likelihood function. Recurrent cases – defined as any case with a period of symptoms in the 8 weeks prior to onset of symptoms (community-onset cases) or hospital admission (hospital-onset cases) – were excluded for the purposes of incidence estimation. The likelihood functions for the incidence of each category (e.g. community-onset hospital acquired) of CDI were normal and independent with mean equal to the reported incidence and standard deviation equal to width of the reported confidence interval divided by $2\times1.96$.

### Proportion of Admissions with CDI

HCUP provide information on hospitalisations in the Unites States stratified by diagnoses. We extracted the total number of hospital inpatient discharges and the number of hospital inpatient discharged where the principal diagnosis was *C. difficile* infection (Diagnoses--ICD-9-CM Codes (ICD9), Principal Diagnosis: 008.45 Int Inf Clstrdium Dfcile) for the year 2014 (the most recent available data at time of extraction) [19]. The likelihood function for the proportion of admissions with CDI was normally distributed with mean equal to the proportion of all discharges where CDI was the primary diagnosis and standard deviation equal to the reported confidence interval divided by $2\times1.96.$

### Alternate fitting assumptions

We considered multiple sets of assumptions when trying to estimate the transmission rates which are best expressed in terms of the transmission parameters giving the transmission rate from each type of colonised individual by setting (hospital or community): β_Comm,Intact,_ β_Hosp,Intact,_ β_Comm,Disrupt,_ β_Hosp,Disrupt_ and β_infant_. Throughout we assumed that β_infant_ was some fraction 0-1 of β_Comm,Disrupt_, however we considered multiple relations between the other parameters.

We tried assuming β_Comm,Intact,_= β_Hosp,Intact,_= 0 and β_Comm,Disrupt,_= β_Hosp,Disrupt_ with generally poor results. Model fit was reasonable when adult colonisation prevalence was low and infants as infectious as adults, however nearly all transmission came from infants in this scenario. For high colonisation prevalence and equal infant infectiousness, though the model fit was still good, this could only be achieved with very high transmission rates in the community and very low (~10%) antibiotics disruption probability and still nearly all transmission came from infants. With lower infant infectiousness in the range 0-0.2 times adult infectiousness, the model had a very poor fit with the incidence in the community being very low (<1/3 of data), and infant colonisation rates being high, transmission rates from adults being excessive (>3) and probability that antibiotics disrupt being very low (<10%).

We tried assuming a single transmission rate β_Comm,Intact,_= β_Hosp,Intact,_= 0 with β_Comm,Disrupt,_ and β_Hosp,Disrupt_ independent. This allowed for arbitrary and independent forces of colonisation in hospital and community and had good model fit for all assumptions. However, in the resulting model nearly all transmission in the community came from infants for most assumed values of infant infectiousness and adult colonisation prevalence. When infant infectiousness was restricted to lower levels (<0.2 β_Comm,Disrupt_), β_Comm,Disrupt_ was up to 50 times greater than β_Hosp,Disrupt_, which is highly implausible.

Finally, our default assumptions (β_Comm,Intact,_= β_Hosp,Intact_ and β_Comm,Disrupt,_= β_Hosp,Disrupt_) resulted in equally good model fit to the previous set of assumptions, but with much more believable transmission rates and proportion of transmission attributable to infants under most combinations of assumptions. However, when infant infectiousness was high and adult colonisation prevalence was low the estimated value of β_Comm,Intact,_ and β_Hosp,Intact,_ were 0, leading to poorer model fit and unbelievably high proportion of transmission from infants and so the result from these extremes were omitted from our sensitivity analyses in the main text and the supplementary figures.

## Further details of model fit

The model fit the data well reproducing the observed age profile of colonisation, immunity, reported incidence of infection, proportion of admissions for CDI (**Supp. Figure 2**). The model was also verified by outcomes not used to fit the model. The reported recurrence proportion is approximately 20% for hospital-acquired cases and approximately 10% of community-acquired cases [20]. We estimated that 18% (range 13-30%) of transmission in hospitals was from patients receiving treatment for CDI. A study using whole genome sequencing to compare isolates from CDI cases in Oxfordshire hospitals estimated that approximately one quarter of cases could be linked to ward-based transmission from another identified symptomatic carrier [21]. Using the same reporting and treatment seeking assumptions used for initial cases and defining a recurrence as a return to symptomatic colonisation within 8 weeks of resolution of symptoms, the model predicted a 19.4% recurrence proportion in hospital-acquired cases (range: 18.5-21.5%) and a 14.0% recurrence proportion in community-acquired cases (range: 13.3-15.8%). Notably, the true recurrence proportions (i.e. without simulating underreporting and misclassification) were higher for both hospital-acquired (24.6-26.8%) and community-acquired (17.8-18.5%) infections. Adlerberth et al [22] found that a third of all infants colonised with *C. difficile* were colonised by the same strain when sampled at least six months later, while the model predicted that 40% (33-42%) of colonised six-month-old infants remain colonised at 12 months of age. A previous estimate of the force of colonisation in a hospital setting [10] derived from the reported risk of colonisation and infection as a function of days of hospital stay [23] was 0.007 day^-1^ (95% CI: 0.004-0.011). In this model the estimated force of colonisation was a little lower: 0.0033 day^-1^ (range: 0.0031-0.0035 day^-1^). However the previous estimate was drawn from a hospital during a period high incidence. In our model, a higher proportion (89.6%, range: 87.9-90.4%) of those classified as hospital-acquired CDI were elderly/immune-supressed compared to cases classified as community-acquired CDI (78.4% range: 77.6-78.6%), in agreement with the observation that community-acquired cases are younger, with fewer comorbidities [24].

## Supplementary Tables and Figures

**Supp. Table 1** Definitions, values and references for all parameters used in the model. All rates are in units of day^-1^. *These parameters were fit to the model, with the range indicating values over sensitivity analysis. †Only these parameters were affected by assumptions around infant infectiousness, being estimated under the assumption that β_Infant_= k × β_Disrupt_ for k in the range 0-1. ‡ These parameter values are the same as our previous model of hospital transmission [10].

| Parameter | Description | Value  Hospital/ Admission | Value  Community/Discharge | References |
| --- | --- | --- | --- | --- |
| *α* | Rate at which patients begin new antimicrobial treatment (proxy for rate of gut flora disruption) | 0.33 | 1.5×10^-3^ | [5,6] |
| *α_U_* | Rate at which patients begin new antimicrobial treatment (proxy for rate of gut flora disruption) for elderly/suppressed | 0.33 | 2.5×10^-3^ | [5,6] |
| *λ* | Rate at which damaged commensal gut flora recovers | 0.03‡ | | [7,8] |
| *λ_o_* | Rate at which damaged commensal gut flora recovers for those with CDI overgrowth | 0.25 | | [9] |
| *γ* | Rate at which *C. difficile* is cleared in those with intact gut flora | 0.023‡ | | [11] |
| *ω* | Rate of *C. difficile* overgrowth in patients with disrupted gut flora | 0.2‡ | | [12,25] |
| *ρ* | Rate at which treatment for CDI is given (hospital) or sought (community) | 1 | 0.22 | [14] |
| *τ* | Rate at which the treatment of patients with  CDI progresses towards resolution | 0.1‡ | | [15,26] |
| *p_t_* | Probability that CDI treatment will remove all *C. difficile* | 0.55‡ | | [10] |
| *q* | Efficacy and coverage of special contact precautions (0: total prevention of transmission; 1: no reduction in transmission) | 0.45‡ | 1 | [13,27] |
| *δ* | Rate of development of immunity in adults | 0.1‡ | | [2,3] |
| *ζ* | Rate at which infants age to develop adult-like gut-flora | N/A | 1/365 | [28] |
| *ψ* | Rate at which people age to suppressed/elderly class | 2.92 × 10^-5^ (Median time: 65 years) | |  |
| *φ* | Death rate | 1.11 × 10^-5^ | | [1] |
| *φ_U_* | Death rate for elderly/suppressed | 1.84 × 10^-4^ | | [1] |
| *N* | Overnight Hospital Beds / Community Size | 150 | 100,000 |  |
| *σ* | Rate at which immunity wanes | 2.9 x 10^-4^ (0.9 x 10^-4^ – 6.9 x 10^-4^) | | * |
| *θ* | Multiplicative factor for colonisation susceptibility of infants | N/A | 1.4 (0.6-4.4) | * |
| *γ_infant_* | Rate at which *C. difficile* is cleared in infants | 0.0020 (0.0020-0.0025) | | * |
| *p_disturb_* | Proportion of antibiotics that disturb gut flora | 0.22 (0.12-0.48) | | * |
| *p_report_* | Proportion of all community-treated CDIs that are reported | 0.63 (0.57-0.76) | | * |
| *β_Disrupt_* | Transmission rate coefficient for colonised adults with disrupted gut flora (due to recent antibiotic exposure) | 0.128 (0.071-0.174) | | *† |
| *β_Intact_* | Transmission rate coefficient for colonised adults with intact gut flora (no recent antibiotic exposure) | 0.019 (0.001-0.026) | | *† |
| *β_infant_* | Transmission rate coefficient from infants | N/A | 0.064 (0-0.174) | *† |
| *ν / κ* | Hospital admission/discharge rate | 2.40 × 10^-4^ | 0.241 | [4] |
| *ν_U_ / κ_U_* | Hospital admission/discharge rate for elderly | 8.06 × 10^-4^ | 0.192 | [4] |
| *ν_CDI_ / κ_t_* | Hospital admission rate for CDI / discharge rate for those receiving CDI treatment | 0.0142  (0.0128- 0.0174) | 0 (Not discharged) | * |


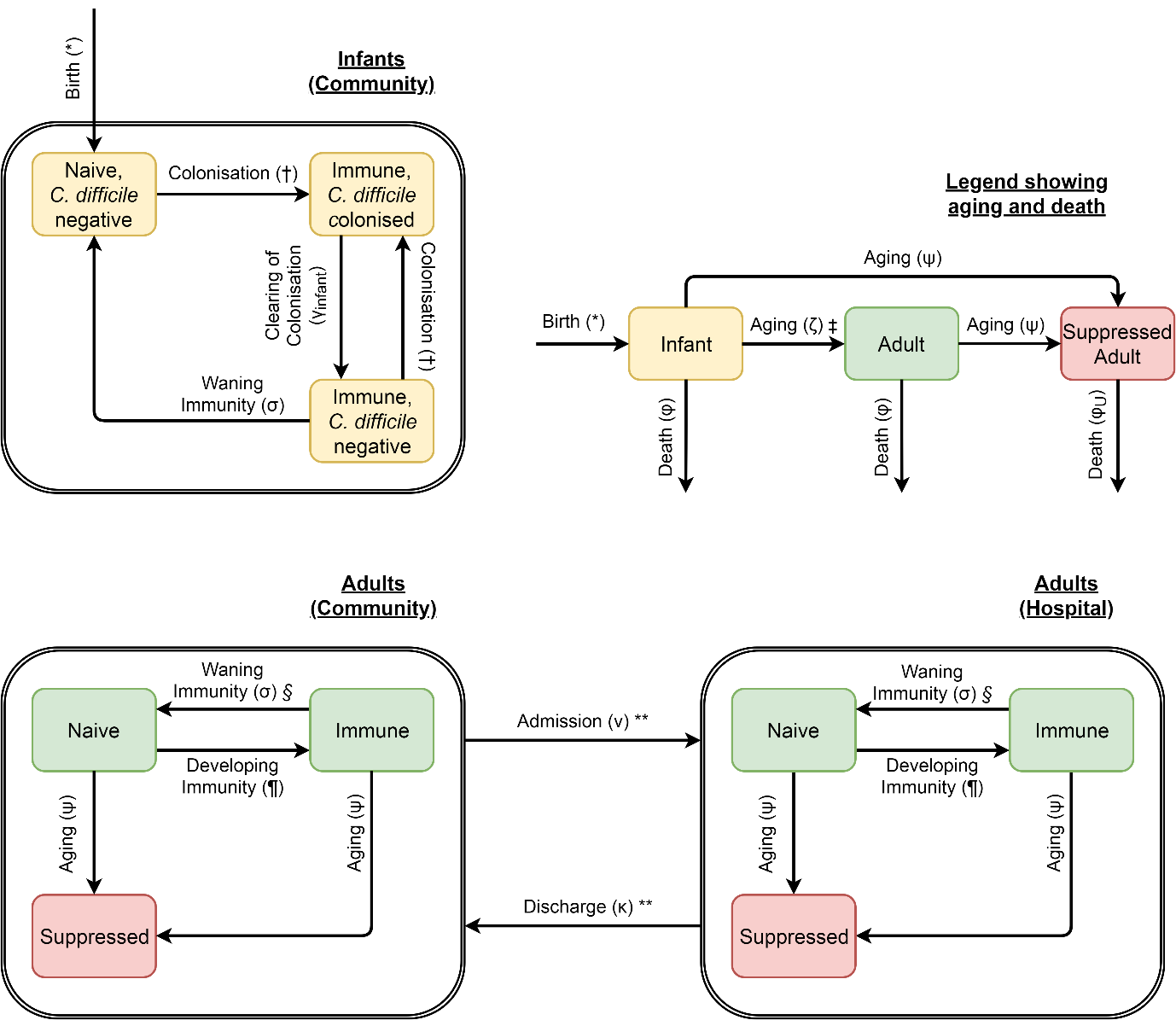


**Supp. Figure 1** Model structure, showing immune states, aging, births, deaths, hospital admission and discharge and infant classes. *Birth rate matches death rate from whole population. †The force of colonisation depends on the number of infectious individuals in the population. Infants are θ times more susceptible to colonisation than adults. ‡Infants retain their immunity and colonisation status when they age to become non-suppressed adults. §Only non-colonised individuals can have their immunity wane. ¶Those with active CDI develop immunity upon recovery. Asymptomatically colonised individuals develop immunity at rate δ. Non-colonised individuals do not develop immunity. **Admission discharge rates vary by immunity and CDI status. Patients receiving treatment for CDI are not discharged and are admitted at a much higher rate.


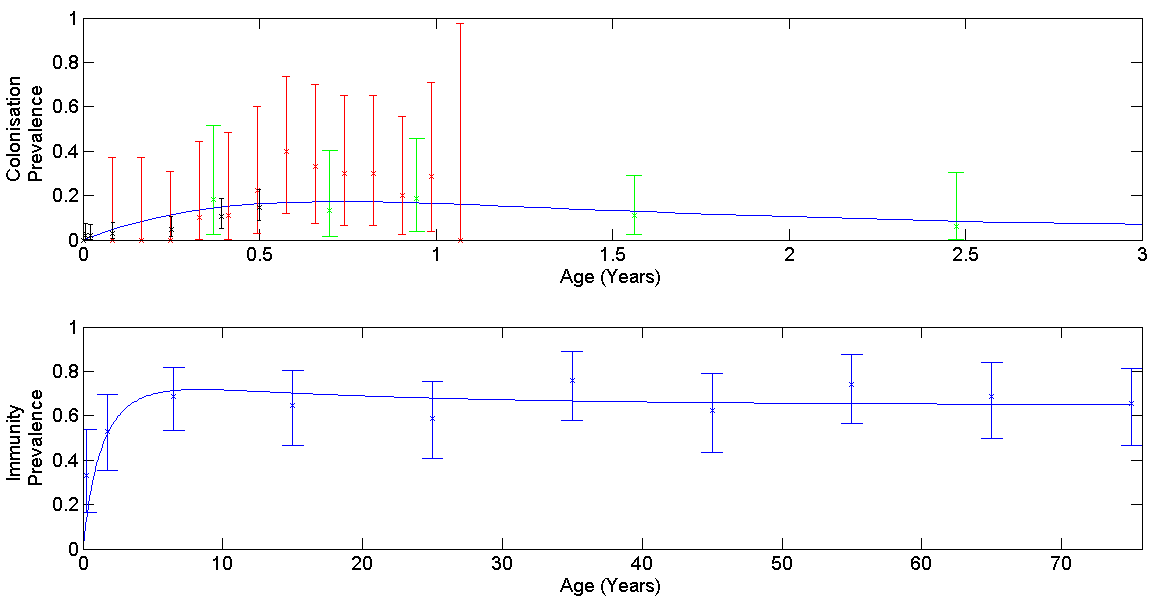


**Supp. Figure 2** Maximum likelihood model fit to infant colonisation data and immunity prevalence data, assuming 5% colonisation prevalence in adults. Blue crosses indicate C. difficile toxin B antibody sero-prevalence [29], green crosses indicate cross-sectional study infants and toddlers in childcare [17]. Red crosses [17] and black crosses [18] indicate longitudinal studies of C. difficile colonisation in infants. All error bars are 95% binomial confidence intervals.


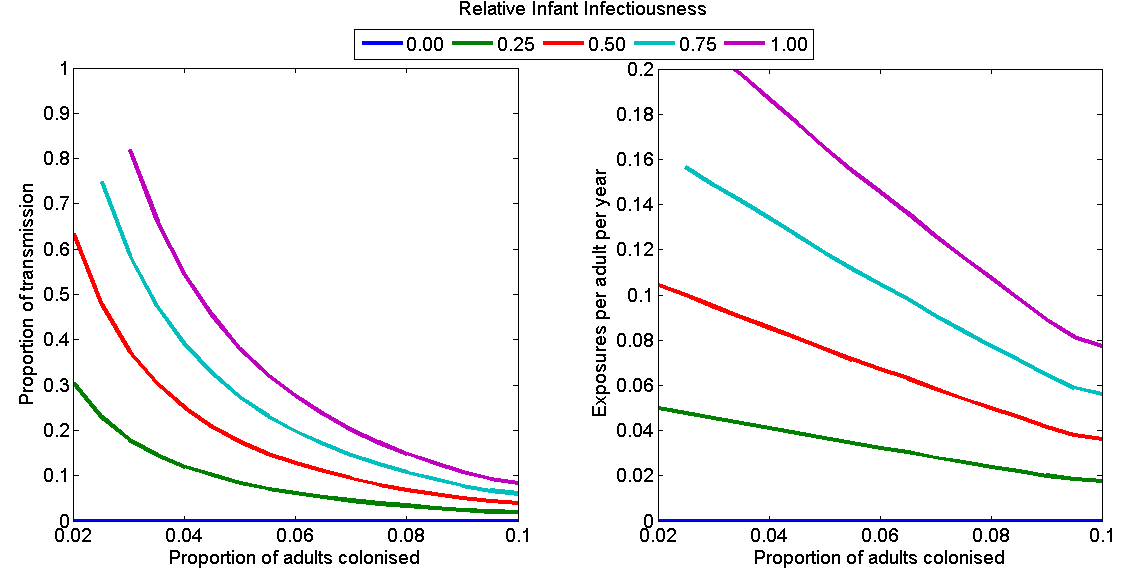


**Supp. Figure 3** C. difficile transmission in the community from infants under various plausible assumptions for the C. difficile colonisation prevalence in adults, and the relative infectiousness of infants as (A) a proportion of all transmission in the community and (B) as rate of exposure to adults in the community.


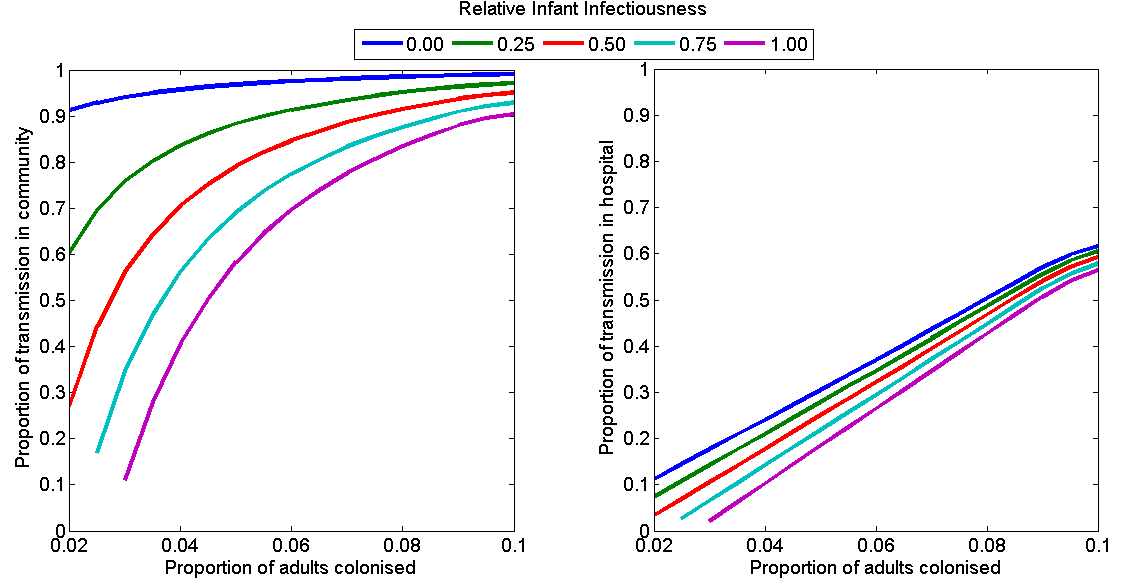


**Supp. Figure 4** C. difficile transmission from colonised adults with intact gut flora under various assumptions for the C. difficile colonisation prevalence in adults, and the relative infectiousness of infants as (A) the proportion of all transmission in the community and (B) the proportion of all transmission in the hospital.

# References

1. **Centers for Disease Control and Prevention - National Center for Health Statistics.** *CDC Wonder: Underlying Cause of Death, Multiple Cause of Death Files, 1999-2016*. (http://wonder.cdc.gov/ucd-icd10.html). Accessed 18 December 2017.

2. **Foglia G, *et al.*** *Clostridium difficile*: Development of a novel candidate vaccine. *Vaccine* Elsevier Ltd, 2012; **30**: 4307–4309.

3. **de Bruyn G, *et al.*** Defining the optimal formulation and schedule of a candidate toxoid vaccine against *Clostridium difficile* infection: A randomized Phase 2 clinical trial. *Vaccine* Elsevier Ltd, 2016; **34**: 2170–2178.

4. **Weiss (Truven Health Analytics) AJ, Elixhauser (AHRQ) A**. *Overview of Hospital Stays in the United States, 2012: Statistical Brief #180*. *Healthcare Cost and Utilization Project (HCUP) Statistical Briefs*. Rockville, MD: Agency for Healthcare Research and Quality, 2014.

5. **MacDougall C, Polk RE**. Variability in rates of use of antibacterials among 130 US hospitals and risk-adjustment models for interhospital comparison. *Infection control and hospital epidemiology* 2008; **29**: 203–11.

6. **Australian Commonwealth Department of Health - Drug utilisation sub-committee (DUSC)**. *Antibiotics : PBS / RPBS utilisation*. Canberra, 2015.

7. **Rafii F, Sutherland JB, Cerniglia CE**. Effects of treatment with antimicrobial agents on the human colonic microflora. *Therapeutics and clinical risk management* 2008; **4**: 1343–58.

8. **Dial S, *et al.*** Patterns of antibiotic use and risk of hospital admission because of *Clostridium difficile* infection. *Canadian Medical Association Journal* 2008; **179**: 767–772.

9. **Bartlett JG**. Treatment of Antibiotic-Associated Pseudomembranous Colitis. *Clinical Infectious Diseases* 1984; **6**: S235--S241.

10. **McLure A, *et al.*** Healthcare-Associated *Clostridium difficile* Infections are Sustained by Disease from the Community. *Bulletin of Mathematical Biology* 2017; **79**: 2242–2257.

11. **Johnson S, *et al.*** Treatment of asymptomatic *Clostridium difficile* carriers (fecal excretors) with vancomycin or metronidazole. A randomized, placebo-controlled trial. *Annals of Internal Medicine* 1992; **117**: 297–302.

12. **Freeman J**. Effects of cefotaxime and desacetylcefotaxime upon *Clostridium difficile* proliferation and toxin production in a triple-stage chemostat model of the human gut. *Journal of Antimicrobial Chemotherapy* 2003; **52**: 96–102.

13. **Muto CA, *et al.*** A Large Outbreak of *Clostridium difficile*–Associated Disease with an Unexpected Proportion of Deaths and Colectomies at a Teaching Hospital Following Increased Fluoroquinolone Use. *Infection Control & Hospital Epidemiology* 2005; **26**: 273–280.

14. **Van Cauteren D, *et al.*** Burden of acute gastroenteritis and healthcare-seeking behaviour in France: a population-based study. *Epidemiology and Infection* 2012; **140**: 697–705.

15. **Leffler DA, Lamont JT**. Treatment of *Clostridium difficile*-associated disease. *Gastroenterology* AGA Institute American Gastroenterological Association, 2009; **136**: 1899–912.

16. **Holst E, Helin I, Mårdh PA**. Recovery of *Clostridium difficile* from children. *Scandinavian Journal of Infectious Diseases* 1981; **13**: 41–5.

17. **Rousseau C, *et al.*** *Clostridium difficile* Carriage in Healthy Infants in the Community: A Potential Reservoir for Pathogenic Strains. *Clinical Infectious Diseases* 2012; **55**: 1209–1215.

18. **Kubota H, *et al.*** Longitudinal Investigation of Carriage Rates, Counts, and Genotypes of Toxigenic *Clostridium difficile* in Early Infancy. *Applied and Environmental Microbiology* 2016; **82**: 5806–14.

19. **Healthcare Cost and Utilization Project (HCUP)**. *HCUP National Inpatient Sample (NIS)*. Rockville, MD: Agency for Healthcare Research and Quality, 2014.

20. **Gerding DN, Lessa FC**. The epidemiology of *Clostridium difficile* infection inside and outside health care institutions. *Infectious disease clinics of North America* 2015; **29**: 37–50.

21. **Walker AS, *et al.*** Characterisation of *Clostridium difficile* hospital ward-based transmission using extensive epidemiological data and molecular typing. *PLoS medicine* 2012; **9**: e1001172.

22. **Adlerberth I, *et al.*** Toxin-producing *Clostridium difficile* strains as long-term gut colonizers in healthy infants. *Journal of Clinical Microbiology* 2014; **52**: 173–9.

23. **Loo VG, *et al.*** Host and Pathogen Factors for *Clostridium difficile* Infection and Colonization. *New England Journal of Medicine* 2011; **365**: 1693–1703.

24. **Khanna S, *et al.*** The Epidemiology of Community-Acquired *Clostridium difficile* Infection: A Population-Based Study. *The American Journal of Gastroenterology* Nature Publishing Group, 2012; **107**: 89–95.

25. **Freeman J**. Comparison of the efficacy of ramoplanin and vancomycin in both in vitro and in vivo models of clindamycin-induced *Clostridium difficile* infection. *Journal of Antimicrobial Chemotherapy* 2005; **56**: 717–725.

26. **Khan FY, *et al.*** Epidemiological features of *Clostridium difficile* infection among inpatients at Hamad General Hospital in the state of Qatar, 2006-2009. *Travel medicine and infectious disease* Elsevier Ltd, 2012; **10**: 179–85.

27. **Jarvis WR, *et al.*** National point prevalence of *Clostridium difficile* in US health care facility inpatients, 2008. *American Journal of Infection Control* 2009; **37**: 263–270.

28. **Jangi S, Lamont JT**. Asymptomatic Colonization by *Clostridium difficile* in Infants: Implications for Disease in Later Life. *Journal of Pediatric Gastroenterology and Nutrition* 2010; **51**: 2–7.

29. **Viscidi R, *et al.*** Serum Antibody Response to Toxins A and B of *Clostridium difficile*. *Journal of Infectious Diseases* 1983; **148**: 93–100.
